# Person identification with arrhythmic ECG signals using deep convolution neural network

**DOI:** 10.1038/s41598-024-55066-w

**Published:** 2024-02-23

**Authors:** Awabed Al-Jibreen, Saad Al-Ahmadi, Saiful Islam, Abdel Momin Artoli

**Affiliations:** 1 Computer Science Department, College of Computer and Information Sciences, King Saud University, 11543 Riyadh, Saudi Arabia; 2 Department of Computer Engineering, Faculty of Engineering, TED University, 06420 Ankara, Türkiye

**Keywords:** Machine learning, Computer science

## Abstract

Over the past decade, the use of biometrics in security systems and other applications has grown in popularity. ECG signals in particular are attracting increased attention due to their characteristics, which are required for a trustworthy identification system. The majority of ECG-based person identification systems are evaluated without considering the health-state of the individuals. Few person identification systems consider person-by-person health-state annotation. This paper proposes a person identification system considering the health-state annotated ECG signals where each person’s beats overlap among variant arrhythmia classes. This overlapping between the normal class and other arrhythmia classes grants the ability to isolate normal beats in the train set from the Arrhythmic beats in the test set. Therefore, this paper investigates the effect of arrhythmic heartbeats on biometric recognition. An effective lightweight CNN based on depth-wise separable convolution (DWSC) is proposed to enhance the performance of person identification for several common arrhythmia types using the MITBIH dataset. The proposed methodology has been tested on nine arrhythmia types and presents how different types of arrhythmia affect ECG-based biometric systems differently. The experimental results show excellent recognition performance (99.28%) on normal heartbeats and (93.81%) on arrhythmic heartbeats, outperforming other models in terms of mean accuracy.

## Introduction

Information security and privacy are considered one of the most important concerns in many organizations, which need to protect their systems, networks, websites, and other services as well as their data. The biometric identification procedure is often recommended as an effective tool for physical and logical access control, as well as surveillance to ensure information security. Biometrics is the measurement of an individual's physiological or behavioral features^1,2^. Biometrics have also been used to lessen hacker assaults in hospitals and other healthcare facilities^3,4^. Due to the widespread use of smart devices like smartphones and smartwatches, biometric recognition has recently gained popularity^3^. However, traditional biometric modalities such as fingerprint, voice, face, iris, and handwriting have found to be vulnerable, as they can be easily replicated and used fraudulently. For example, a fingerprint can be recreated by latex, the face is vulnerable to artificial masks, an iris can be faked using contact lenses with copied iris features printed on it, and the voice can be mimicked easily. Some of them might be "lost" following severe injuries and trauma or drastically changed by plastic surgery^5^. Additionally, these biometric systems' performance is not always satisfactory in a number of applications^5^. ECG signal also known as heartprint^6^ is an emerging biometric modality employed to improve security. This modality has the inherent liveness characteristic as they can only be obtained from an alive person. As the heart is confined in the structure of the body, ECG signals are difficult to be fabricated or altered unlike face, finger, or iris biometrics^4^. Moreover, good quality ECG signals can be easily be captured from fingers^6^. All these make it a more acceptable and secured biometric modality.

There are numerous existing ECG-based biometric systems with excellent performance on regular ECG signals^7,8^. These systems either developed their own deep learning-based systems or used the transfer learning method with pre-trained CNNs. These systems perform differently depending on the health state. On arrhythmic ECG signals, the same systems perform less well, than regular ECG signal- based recognition system performance. There has not been much research or analysis done on the arrhythmic ECG signal's structure. Authors in^9^ test their multi-layer perception network model on arrhythmic signals. The most recent advancement of ECG-biometric systems that have been proposed is based on deep-learning techniques; nevertheless, there is no detailed investigation on whether a deep-learning-based model is more suitable for arrhythmic ECG signals.

To the best of our knowledge, no previous study has tested the arrhythmic versus normal heartbeats of the same patient separated into train and test sets to proposed a complete analysis of the impact of different types of arrhythmia on the ECG-biometric system. As a result, our research suggests a thorough examination of how various types of arrhythmia affect an ECG-based biometric system. A deep-learning, beat-labeled ECG-based biometric system is also proposed in this study. The research's contribution includes the following:

- A deep CNN model is designed based on the depth-wise separable convolution (DWSC) operation with a good trade-off between the low parameter complexity and the high representation capacity for achieving good person identification performance on ECG signals.
- Using the designed CNN model, a robust ECG-based identification system is developed to reduce the adverse impacts of four different types of arrhythmia.
- We isolate the arrhythmic heartbeat set and the normal heartbeat set of the same individuals in train and test sets in order to analyze the effects of arrhythmia on the person identification system. To the best of our knowledge, no person identification system isolates the heartbeats of the same individual based on the heart-beat health-state annotation in the training and test sets.
- We found a relationship between the heartbeat's health state and the performance of the ECG-based person identification system: if the person does not suffer from cardiac disease, the performance is excellent.
- Experimentally, we show a negative impact of arrhythmia on the performance of the person identification system. The severity of the adverse effects varies depending on the type of arrhythmia.

The rest of the paper is structured as follows: Next section presents the related work. Then the proposed model is described. The following section presents results, the evaluation criteria, and the implementation details. In the conclusion, the results are highlighted and future research is underlined.

## Related work

ECG identification systems are built based on either hand engineering/machine learning methodologies or deep learning methods. Many ECG-based recognition systems have been proposed since 2001 when ECG-signal was first introduced as a biometric modality^10^. Most of the state-of-art studies proposed solutions based on Deep Learning models for ECG Biometric systems. CNN and BLSTM are the most applied models in ECG-based biometric systems that perform the best. Yazhao Li et al.^11^ suggest a CNN architecture for identification and feature learning process. Eko Ihsanto et al.^12^ suggested Residual Depth-wise Separable Convolutional Neural Network (RDSCNN) deep learning approach for ECG-based recognition. Zhidong Zhao et al.^13^ test their suggested system using various dataset scenarios, regular dataset and the Atrial Fibrillation dataset were both used for system evaluation. The results of the suggested method are good, coming in at 99% and 98%, respectively.

There are three algorithms where biometric performance is evaluated separately between healthy and arrhythmic subjects. Authors in^14^ suggested a methodology and tested it and compare it versus other two methodologies. They test the three techniques on 112 PTB subjects (98 subjects have arrhythmia beats and 14 healthy subjects.).The first methodology is proposed in^14^, the Pulse Active transform (PAT). PAT is a transformation technique; it decomposes a signal into a series of related cyclic triangular waveforms (pulse active feature sets). Matching scores are generated by the Euclidean Distance as the distance measure. The second technique is proposed in^15^, where authors proposed a set of extensive ECG features proven to be invariant to anxiety state. These descriptors are the fiducial points; they contain physiological information of the heart. They develop filtering technique that merges heuristic and quantitative information. The third technique is proposed in^10^ Authors use about twenty-one fiducial features from single lead. These features include amplitudes, waves and segments. Soft independent modeling of class analogy (SIMSA model) is applied for classification. The first step in SIMCA is applying PCA for each class to express data variance. Next step is building models of different classes using measurement sets. The proposed methods in^10^ and in^15^ are applied in^14^ on same data and the results are shown in Table 1. It can be concluded in the results that in all the three different proposed methodologies, Healthy subjects score better identification performance versus Arrhythmic subjects. It also shows that PAT outperforms other two other methodologies.

**Table 1:** ECG based biometric systems on normal and arrhythmic subject.

Study	Dataset	The proposed model	Considered health-state annotation	Person health state	Performance
Biel et al.^10^	PTB	21 Fiducial Features, PCA, SIMCA	Person by Person	Healthy Unhealthy	85.4 76.3
Israel et al.^15^	PTB	Fiducial Features, LDA	Person by Person	Healthy Unhealthy	81.4 74
Safie et al.^14^	PTB	Pulse Active Transform PAT	Person by Person	Healthy Unhealthy	95 85
Huang, et al..^16^ Thentu, et al.^17^ LYNN, et al..^18^ Xin Liu, et al.^19^	MITBIH	Deep Learning Techniques	Health-state not considered	Mixed	99.98 EER = 0.13 98.4 99.77

Above review shows that Deep learning methods scores higher performance than hand engineering methods. It is noted that the same system performs differently based on the data health state (normal/arrhythmic). Researches have examined biometric systems using both normal and arrhythmic datasets, but none of them have provided a thorough examination of the various types of arrhythmic signal and how they impact system performance negatively. Moreover, no ECG-based Biometric system has tested Arrhythmic beats versus Normal beats for same person in Test/Training set.

## Methods

**Figure 1:**
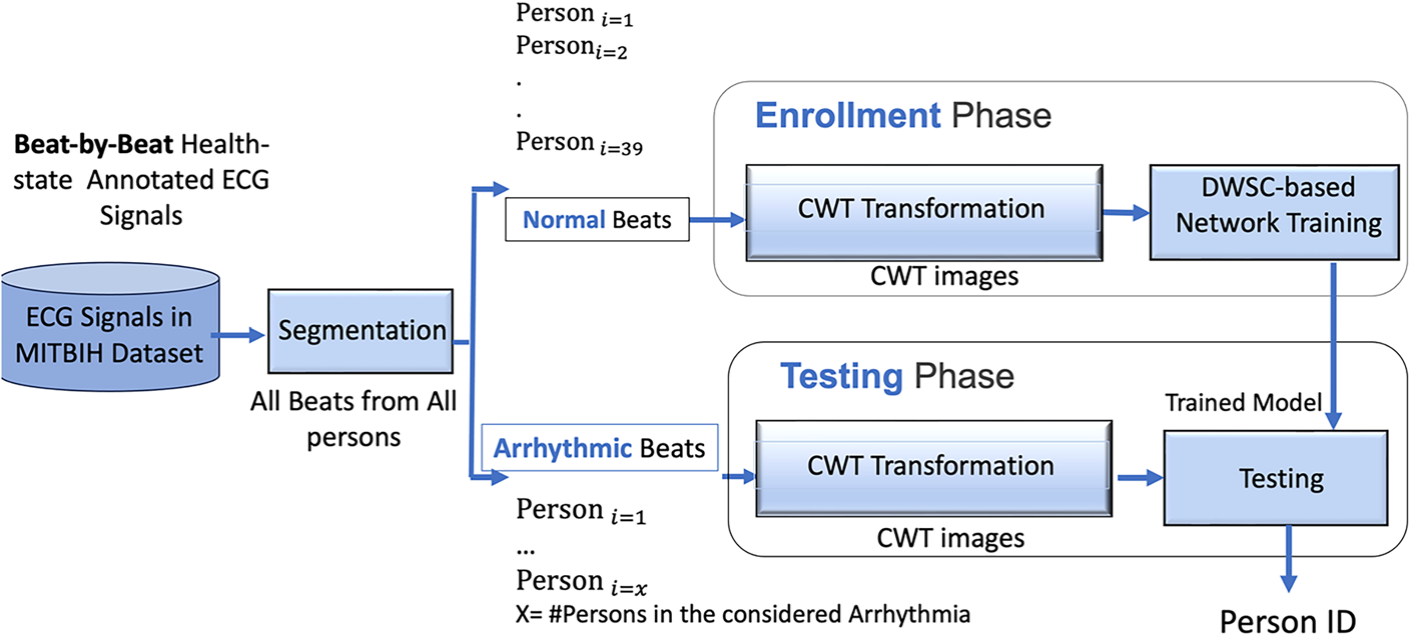
Block diagram of the proposed method.

Deep neural networks make it possible to thoroughly examine data sets without requiring in-depth domain expertise or analytical prowess. Deep learning-based methods automatically learn features, unlike traditional biometric methods that require manual feature extraction. Our goal is to examine and develop the best segmentation and deep learning model for identification system using arrhythmic ECG signals. Figure 1 presents the block diagram of the proposed methodology. Convolution Neural Network (CNN) is proposed in this study as both feature learner and classifier. The details of the proposed CNN architecture are presented in the following subsection.

### Change of heartbeat morphology due to arrhythmia

**Figure 2:**
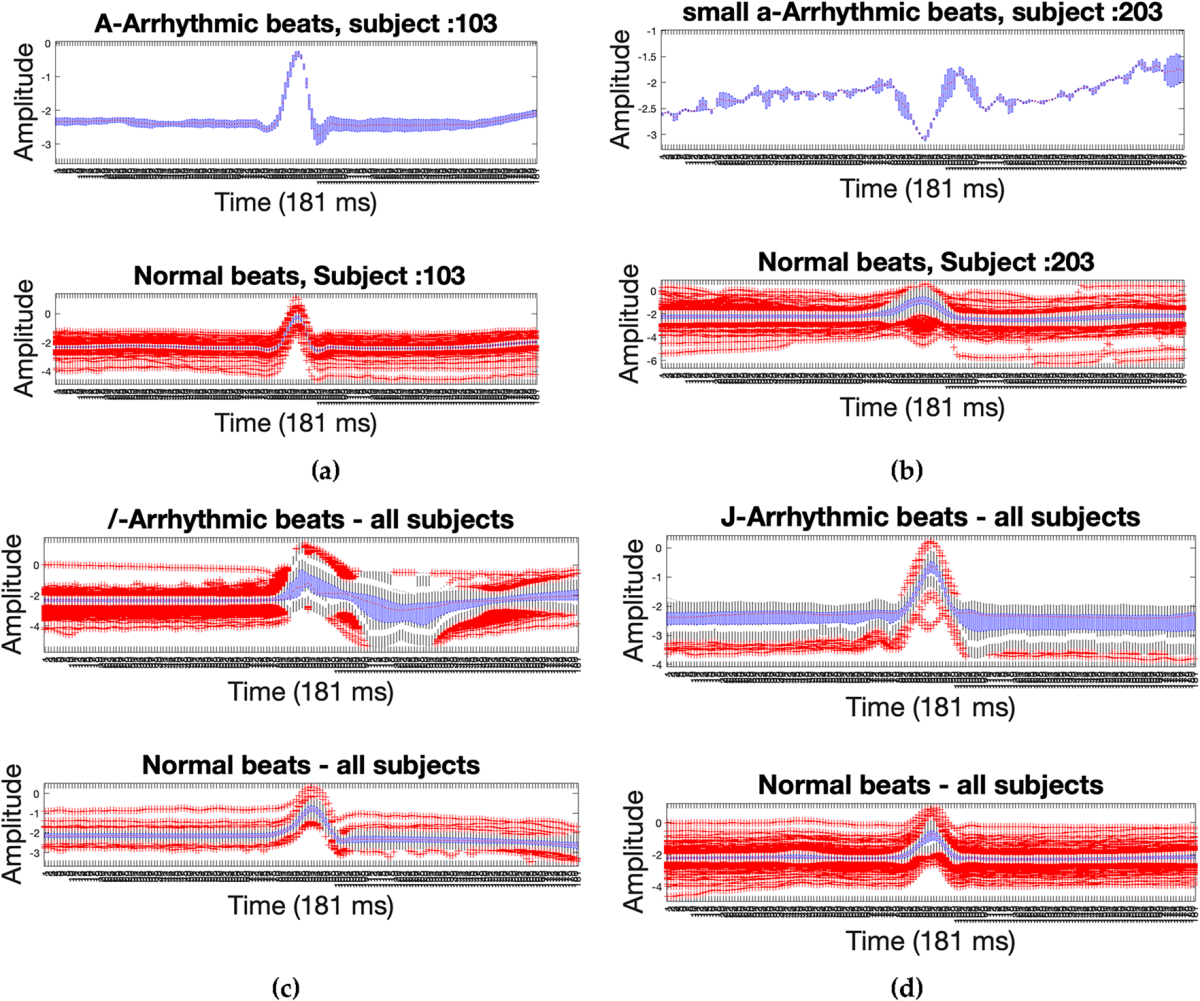
Heartbeat morphology of ECG signal around R-peak for arrhythmic (up) vs normal (down) beats for subjects of MIT-BIH dataset: (**a**) Atrial premature beats (A-arrythmia) for subjects# 103; (**b**) Aberrated atrial premature beat (small a-arrythmia) for subjects# 203; (**c**) Paced beat (/—Arrhythmia)) for all common Subjects; (**d**) Nodal (junctional) premature (J- Arrhythmia) for all common subjects. (**a**) and (**b**) subfigure shows the intra-individual similarities; inter-individual differences among the individuals can be observed from these two subfigures.

Arrhythmia, or an abnormal rhythm of the heart, changes the morphology of heartbeats. There are various types of arrhythmia, and each type has its own unique pattern^20^. Therefore, the change in morphology depends on the type of arrhythmia. Due to the morphological change of the ECG signal, it has an impact on biometric systems that use an ECG as a basis. Figure 2a,b depicts the morphological changes of individual subject for different arrhythmias in MIT-BIH dataset^20^. In Fig. 2c,d show morphological changes collectively for all subjects for variant types of arrhythmias in MIT-BIH dataset.

The statistical summary of the ECG beat samples is represented in Fig. 2, which provides the following features:

- The distance between each box's top and bottom is called the interquartile range.
- The center red line denotes the sample median for each box.
- Box whiskers are lines that extend above and below the box. The whisker length is measured from the end of the interquartile range to the furthest observation.
- The outliers are those that fall outside of the whisker length. An outlier is a value that is more than 1.5 times the interquartile range from either the top or bottom of the box. An outlier is indicated by a red plus sign.

### Data preprocessing approach

The ECG signal is a recording of the electrical activity of the heart that represents the regular pattern of heartbeats made up of P-QRS-T waveforms. Some preprocessing is required to get the ECG signal ready for the following stages, including signal segmentation to have the most relevant segments. In this study, beats are segmented based on the locations of the R-peaks recorded the index-annotation file in the MIT-BIH dataset. The R-peak is simpler to detect because of its lengthy duration and high amplitude^21^. We apply the segmentation technique on 181 ms-wide window (0.5 s) following the research in^22–24^ where this signal length gives the best result on the PTB arrhythmic dataset, with R-peak at its central point.

The adopted preprocessing approach in this research is inspired by the information given in^25–27^. In this research, R-peak position is basically centered in the beats segmentation process. However, R-peak is later re-positioned according to ECG intervals. The authors in^27^ state that the PQ interval consumes 80 ms, which is roughly 22.22% of the ECG beat. QR-interval consumes 120 ms, roughly 33.33% of the whole beat. T-wave consumes 160 ms, which is roughly 44.44%. Therefore, T-wave consumes about double of the part consumed by P-wave. In^25^, authors present similar calculations, suggesting that QT-interval consumes about three times what PQ-interval consumes. it is also shown that the PQ interval ranges from 0.6 to 0.79, which is roughly 25.6% of the whole ECG beat. QT-interval consumes from 0.81 to 1.25 (about 0.44) which is 31.43% of the whole ECG beat in^26^. These calculations suggest that R-peak is not supposed to be in the center of the ECG segment. The adopted approach is to place the R-peak before the center of the ECG segment. This approach has increased the recognition performance for four types of arrhythmic heartbeats.

**Figure 3:**
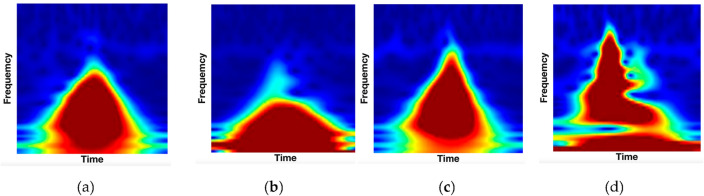
The CWT transformation images of ECG heartbeats from the MITBIH Dataset^28^ of ECG Beats for: (**a**) ECG Normal Beats; (**b**) Premature ventricular Contraction (V- Arrhythmia); (**c**) Atrial premature beat (A-Arrhythmia); (**d**) Paced beat (/—Arrhythmia); Heartbeats morphology differences among the three types of Arrhythmias can be observed from the three subfigures.

After beat segmentation, ECG signal is transformed via a time–frequency transformation technique into a two-dimensional image. Scalogram refers to this representation, which is made up of the absolute value of the signal's CWT coefficients. Figure 3 demonstrates the CWT transformation of ECG normal beats (right) and ECG specific-arrhythmia-type beats (left).

### Developing deep learning-based ECG biometric model

Deep learning has become the most widely used model in ECG biometric systems. Different deep learning architectures adopted in ECG biometric systems have been investigated in^29^. Deep learning-based systems typically outperform traditional hand-engineered and machine-learning-based systems. Deep learning-based system adaptation can be accomplished in two ways. The first involves starting from scratch and training a new network. The second method uses Transfer Learning, which enables us to train an already-built and previously trained CNN before on our dataset. High model efficiency necessitates a straightforward architecture with fewer parameters. By using a sufficient number of training samples and suitable numbers of parameters, over-fitting and under-fitting issues are easy to be avoided. The following subsection present an explanation of the proposed CNN and its components.

#### Depth-wise separable convolution (DWSC)

**Figure 4:**
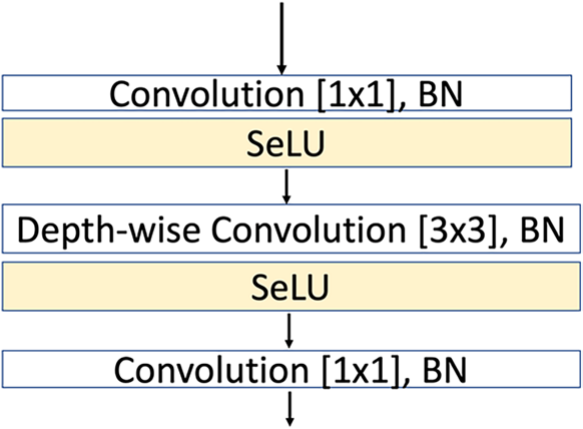
Basic building block (DWSC).

The proposed CNN include variant types of layers: convolution layer, batch normalization layer, and activation layer. Convolutional layers are the basic layer where the convolutional process could be standard or depth-wise convolution. In depth-wise convolution, channel-wise and spatial-wise computations are fulfilled in one step. The approach proposed in^30^ served as the main inspiration for the suggested model. The authors in^30^ propose a depth-wise separable convolution architecture. Figure 4 illustrates the basic building block of the proposed model. The basic block of the proposed model contains depth-wise separable convolutions (DWSC) that were proposed in^30,31^. DWSC consists of two processes: depth-wise and point-wise convolution, Depth-wise convolution convolves each input feature map with a single convolutional filter. Then, point-wise convolution is used to make a linear combination of depth-wise convolution outputs.

The first step in the proposed model is applying a standard convolutional filter K to the input feature map F to produce the output feature map ${\text{\rm O}}$:

1 $${\text{O}}_{k,l,n} = \mathop \sum \limits_{i,j,m} K_{i,j,m,n} { }.{ }F_{k + i - 1,l + j - 1,m}$$

Then, depth-wise separable convolution is applied by: (1) applying a 3 × 3 depth-wise convolution $\ddot{K}$ to Each input channel,

2 $$\hat{\rm O}_{k,l,m} = \mathop \sum \limits_{i,j} \hat{K}_{i,j,m} \cdot F_{k + i - 1,l + j - 1,m}$$

**Figure 5:**
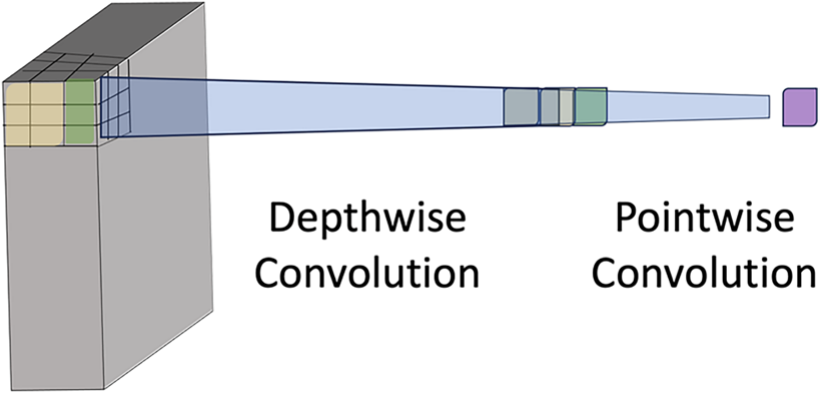
Depth-wise Separable Convolution.

(2) Combining the depth-wise output by applying 1 × 1 pointwise convolution $\ddot{K}$,

3 $${\rm O}_{k,l,n} = \mathop \sum \limits_{m} \tilde{K}_{m,n} \cdot \hat{\rm O}_{k - 1,l - 1,m}$$

where: m and n is number of the input and output feature maps respectively. Depth-wise Separable Convolution is presented in Fig. 5. The architecture of the proposed CNN model is shown in Table 2.

**Table 2:** Architecture of the proposed CNN model.

Layer/block	Data size × Filter size * #Filters/nodes	Convolution stride
Input layer	$32^{2} \user2{ } \times 3\user2{ }$	–
Basic block 1	$32^{2} \user2{ } \times \user2{ }3\user2{ } \times 32$	2
Basic block 2	$16^{2} \user2{ } \times \user2{ }3 \times \user2{ }64$	1
Basic block 3	$16^{2} \times 3\user2{ } \times \user2{ }128$	2
Basic block 4	$8^{2} \user2{ } \times \user2{ }3\user2{ } \times 256$	1
Basic block 5	$8^{2} \user2{ } \times \user2{ }3\user2{ } \times \user2{ }512$	1
Average pooling	$1^{2} \user2{ } \times 512$	–
Fully connected	$1^{2} \times 200$	–
Elu activation	$1^{2} \times 200$	–
Fully connected	$1^{2} \times 39$	–
Softmax	$1^{2} \times 39$	–
Classification	$1^{2} \times 39$	–

#### Activation function

The proposed model is incorporated with nonlinearity through two Activation Functions: (1) Scaled Exponentially Linear Units(SeLU), proposed in^32^, Where $\uplambda$ =1.0507 and $\alpha$ =1.6733

4 $$SeLU_{\left( x \right)} = {\uplambda }\left\{ {\begin{array}{*{20}c} x & {if \;x > 0} \\ {\alpha (e^{x} - 1)} & {otherwise} \\ \end{array} } \right.$$

SeLU non-linearity is one of ELU’s variants. SeLU’s important property is introducing self-normalizing^33^, it is applied in the first two blocks in the proposed model. (2) Gaussian error linear units (GeLU) activation is applied in the next following blocks to introduce regularizers, such as dropout with the activation function. GeLU procedure is performed by the following formula, where $x$ is the input:

5 $$GeLU_{\left( x \right)} = \frac{1}{2} \times \, \left( {{1} + \tanh \left[ {\sqrt {\frac{2}{\pi }} \left( {x + 0.044715x^{3} } \right) } \right]} \right)$$

Instead of using gate inputs by their sign, like in ReLU, we adopt the GeLU activation function to introduce the nonlinearity weighted inputs computed by the inputs value. The experiments in^34^ show that GeLU matches or exceeds models with ReLUs or ELUs within variant fields such as NLP fields, computer vision, and automatic speech recognition. The Gaussian distribution function computes the cumulative distribution function (CDF)^34^.

## Experiments and Results

Arrhythmic datasets are provided by PhysioNet^35^ for a patient population. MIT-BIH^28^ includes 47 participants total—25 men, ages 32 to 89, and 22 women, ages 23 to 89. A detailed statistic is presented in Table 3. This study focuses on this dataset because it is the only one that offers beat-labeling (A or N) for every subject. Every other dataset with an arrhythmia offers a subject-labeling. Therefore, the MIT-BIH dataset is the only one of these datasets that is beat-annotated rather than person-annotated. As a result, the MIT-BIH dataset is the main focus of this study.

**Table 3:** The statistical Information of the MITBIH Dataset.

Beats health state	#Persons^a^	#Persons with normal beats
Normal (Healthy)	39	39
Premature Ventricular Contraction (V- Arrythmia)	36	29
Atrial Premature (A- Arrhythmia )	36	22
Fusion of ventricular and normal (F-Arrhythmia )	16	2
Nodal (junctional) escape (small-j Arrhythmia))	4	3
Aberrated atrial premature (small a)	6	6
Fusion of paced & normal beat (small-f)	3	3
Isolated QRS-like artifact (|—Arrhythmia )	20	16
Nodal (junctional) premature (J-Arrhythmia)	6	5
Right Bundle Branch Block (R-Arrhythmia)	6	2

^a^In The MITBIH Dataset, PersonID may overlap among multiple Arrhythmia classes.

## Evaluation

The number of samples TP, FP, TN, and FN is computed by the confusion matrix, where T is true, F is false, P is positive, and N is negative. Evaluation criteria are computed using these sample numbers as follows^33^:

6 $${\text{Accuracy }} = \frac{TP + TN}{{TP + FP + FN + TN}}$$

7 $${\text{Specificity }}\left( {{\text{TNR}}} \right) \, = \frac{TN}{{FP + TN}}$$

8 $${\text{Recall }}\left( {{\text{TPR}}} \right) = \frac{TP}{{TP + FN}}$$

9 $${\text{Precision }} = \frac{TP}{{TP + FP}}$$

10 $${\text{F1}} - {\text{Score}} = { 2} \times \frac{{{\text{Precision }} \times {\text{ Recall }}}}{{{\text{Precision }} + {\text{Recall }}}}$$

The evaluation of different pre-trained CNNs identification systems for regular beats was the first step in this study. The top results in the research and the complexity of the networks are taken into consideration when selecting the five pretrained networks. Table 4 demonstrates that the proposed model achieves recognition rates better than or competitive to the state-of-the-art networks, including Xception^36^**,** Shufflenet^37^ and MobilenetV2^38^, which provided the best outcomes of the MITBIH dataset on regular beats. The table also shows whether the network is a Directed Acyclic Graph (DAG) or a linear architecture network.

**Table 4:** Deep network models parameters, time consumed and recognition rate on regular heartbeat.

Model	# Learnable parameters	#Layers	Training time (in hours)	#Epocs for training	Image size	Recognition rates on regular heartbeats	CNN architecture
Inception^39^	23.9 M	315	52.4	7	${299}^{2}$	96.97	DAG
Xception^36^	22.9 M	171	67.3	4	${299}^{2}$	99.32	DAG
Squeezenet^20^	1.2 M	68	3.1	5	${227}^{2}$	98.68	DAG
Googlenet^40^	6.9 M	144	11.7	15	${224}^{2}$	98.50	DAG
MobileneV2^41^	3.5 M	154	5.3	5	${224}^{2}$	99.17	DAG
Shufflenet^37^	1.4 M	172	2.5	5	${224}^{2}$	99.29	DAG
Proposed Model	**665 k**	**46**	**58 Min**	10	${32}^{2}$	**99.28**	**Linear**

M: Millions, K: Kilos.

Significant values are in bold.

**Figure 6:**
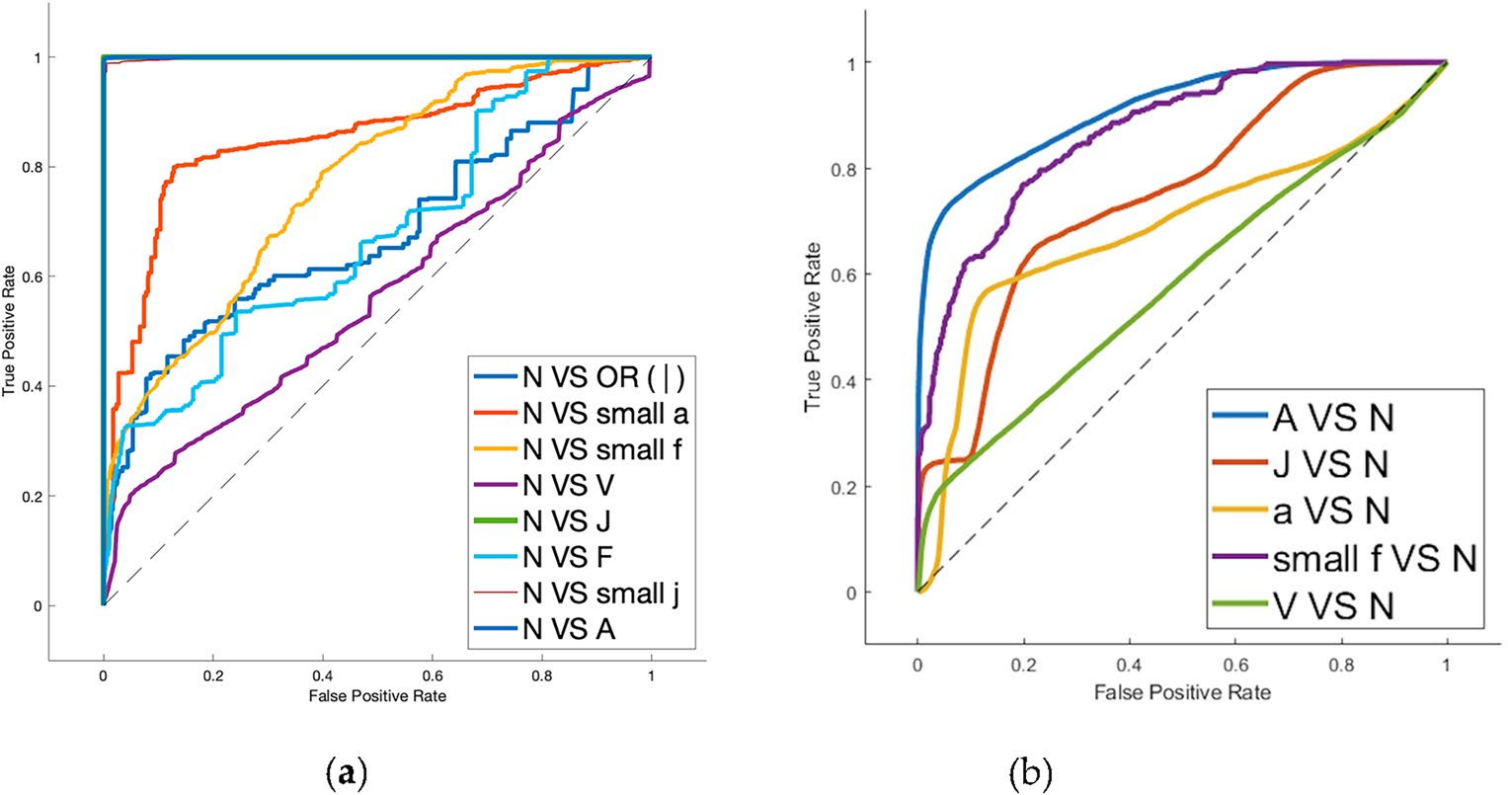
ROC Curves of the validation curve on person identification system evaluated on the MIT-dataset for: (**a**) Normal beats (training set) versus one arrhythmia type beats (test set); (**b**) one arrhythmia type (training set) versus normal beats (test set).

**Figure 7:**
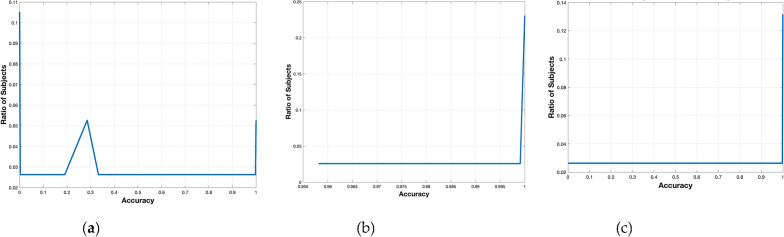
Cumulative distribution plot (CDF) in Person identification on the MITBIH dataset for: (**a**) normal versus all arrhythmic beats; (**b**) normal vrsus normal; (**c**) all-arrhuthmic beats versus all-arrhythmic beats.

**Figure 8:**
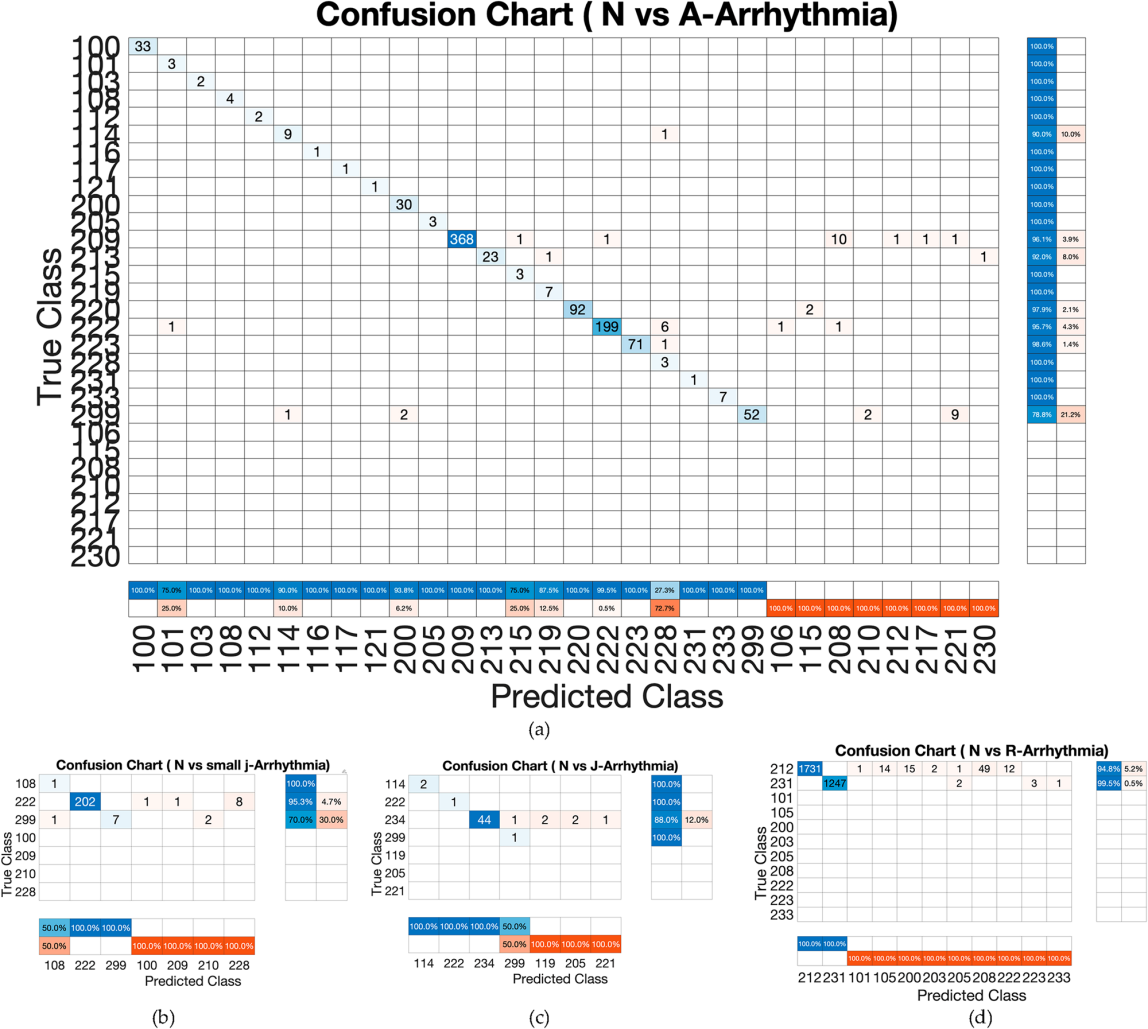
Confusion charts of the proposed methodology results for the four considered arrhythmia. The last two columns are the true positive and false negative rates respectively. The last two rows are the false positive and true neagtive rates respectively.

Exploring and evaluating the various ECG biometric system architectures for both normal and arrhythmic datasets will help us achieve our first goal. Therefore, as a second step, we compare different arrhythmia types’ negative impact on ECG biometric systems in Table 5, where Mobilenet^41^ is tested on each arrhythmia type separately, isolating the same subject’s arrhythmic heartbeats from his/her Normal heartbeats in the test set or train set. ROC curves are shown in Fig. 6 while Fig. 7 shows the cumulative distribution plot (CDF). Figure 8 shows the confusion charts for the four Arrhythmia types. Table 5 also shows the case where both the train and test sets contain only arrhythmic beats, the results are excellent (97.67%). This is because the network trains and learns the characteristics and morphologies of the same type of beats in the test set.

**Table 5:** ECG-based identification performance of variant types of arrhythmia. N: normal beats. A: arrhythmic beats.

Arrhythmia type	Evaluation metrics									
	Train set (N) versus test set (A)					Train set (A) versus test set (N)				
	Acc	Prec	Sens	Rec	F1	Acc	Prec	Sens	Rec	F1
All arrhythmia types	31.79	0.23	31.68	99.9	0.46	48.16	5.36	47.5	96.2	0.10
Premature ventricular Contraction (V-Arrythmia)	18.5	*	18.5	100	*	19.27	17.3	79.9	100	0.3
Atrial premature (A-Arrhythmia )	93.64	36	93.8	99.5	0.53	64.16	12.2	63.8	96.5	0.22
Fusion of ventricular and normal (F-Arrhythmia )	60.71	0.32	60.8	99.6	0.01	31.29	0.96	31	98.7	0.02
Nodal (junctional) escape (small-j Arrhythmia))	80.7	17.3	79.9	100	0.3	80.7	17.3	79.9	100	0.3
Aberrated atrial premature (small a)	25.3	*	25.3	100	*	52.16	19.8	45.8	99.9	0.33
Fusion of paced & normal beat (small-f)	61.61	17.3	79.9	100	0.3	–	–	–	–	–
Isolated QRS-like artifact (|—Arrhythmia)	57.6	*	57.6	*	*	–	–	–	–	–
Nodal (junctional) premature (J-Arrhythmia)	59.26	*	59.26	100	*	49.46	9.95	46.8	98.8	0.18
Right bundle branch block (R-Arrhythmia)	93.57	*	93.57	100	*	–	–	–	–	–

*These values are affected by the zero values in confusion matrix.

## Results

At first, we tested the effect of different components of the proposed model on the performance of recognition using arrhythmic heartbeats. it is observed from Table 4 that the four arrhythmia types (J, A, R, & j) has less impact on the recognition performance. In general, the performance is excellent if the person does not have an arrhythmia as presented in Table 3. However, the performance is worse if the person has arrhythmia. Depending on the type of arrhythmia, the performance varies. For instance, of all arrhythmias, atrial premature (A-arrhythmia) scores very swell. This is because, as seen in Figs. 2a and 3b, A-arrhythmia does not significantly alter the form of the heartbeat. In contrast with the occurrence of premature ventricular contraction arrhythmia (V-arrythmia) which severely changes the morphology of heartbeats, as shown in Fig. 3a.

Depth-wise separable convolution (DWSC) has been applied in several studies related to image processing tasks^30,42–44^. Authors in^30^ evaluate their model, which is based on DWSC on ImageNet and CIFAR100 datasets and show that the depth-wise approach scored the highest score, requiring much lower parameters compared with the other state-of-the-art approaches.

We evaluated the proposed model and other six pretrained networks on Normal betas (as a Train set) vs. Arrhythmic Beats (J, A, R, & J) as a test set and results are presented in Table 6. In addition, we tested these networks and the proposed model on Normal beats (as a Train set) vs. Arrhythmic Beats (All arrhythmia types) as a test set, and results are presented in Table 6. In addition, to explore DWSC effects, we tested the proposed model with DWSC versus the proposed model with standard convolution (SC) instead. Results in Table 6 shows that the proposed model with DWSC score competitive accuracy with about 5 × lower number of learnable parameters than the proposed model without DWSC operation.

**Table 6:** Recognition accuracy of normal (training set) versus arrhythmic beats (test set) with and w/o DWSC.

Model	Activation	Accuracy (%)			
		W/o DWSC (SC-model)		With DWSC	
		(J, A, R, & j) Arrhythmia	All Arrhythmia	(J, A, R, & j) Arrhythmia	All Arrhythmia
Googlenet^40^	**ReLU **^39^	57.32	27.30	NA	NA
Inception^39^	**ReLU**^20^	78.45	26.65	NA	NA
Squeezenet^20^	**ReLU **^40^	69.41	23.09	NA	NA
Xception^36^	**ReLU **^36^	NA	NA	95.53	30.28
Shufflenet^37^	**ReLU**^41^	NA	NA	94.41	28.69
MobileneV2^41^	**ReLU **^37^	NA	NA	95.07	29.56
Proposed Model	**SeLU/GeLU**	**96.08**	30.11	**96.22**	29.17

Significant values are in bold.

The main contribution in this study is the results shown in Table 7 where the proposed model improves performance of person identification system with the four types of arrhythmias. Table 7 shows that the proposed model outperforms other models in term of the mean accuracy. Considering the proposed model’s simplicity (664 k learnable Parameters) and other models’ complexity (2 to 20 million learnable parameters, this is another achievement of this study in addition to highest mean accuracy. The proposed model is evaluated on normal heartbeats and achieves a good recognition performance (99.6%) presented in Table 8.

**Table 7:** Identification results of the proposed methodology compared with other models (N vs. A).

Arrhythmia Type	Inception	Xception	Googlenet	Squeezenet	Shufflnet	Mobilenet	Proposed methodology
Atrial Premature (A- Arrhythmia )	90.82	86.86	70.43	81.98	91.34	93.64	**95.41** $\pm$ 0.2093
Nodal (junctional) escape beat (small-j Arrhythmia)	94.62	91.93	87.36	89.73	**95.52**	90.84	**94.17** $\pm$ 0.2348
Nodal (junctional) premature ( J-Arrhythmia)	61.11	**90.74**	46.30	54.54	61.11	59.26	88.89 $\pm$ 0.3172
Right Bundle Branch Block (R-Arrhythmia)	59.17	**98.57**	54.32	64.21	**95.87**	**93.57**	96.75 $\pm$ 0.1773
Mean accuracy	76.43	**92.03**	64.6	72.62	85.96	84.33	**93.81**

Significant values are in bold.

**Table 8:** Performance comparison of the proposed model and other models in the MITBIH Dataset.

Refs	Preprocessing, feature extraction and classification	Acc
^45^–2019	Segmentation & Convolutional Neural Network	98.9
^18^–2021	Heartbeat Segmentation and RNN	98.4
^46^–2022	Wavelet transform & Kalman filter & (IPSO-SVM)	95.17
^47^–2023	Auto Correlation/Discrete Cosine Transform, Feature reduction method & City Block	99.4
Proposed	Heart Beat Segmentation, CWT & CNN	99.58

## Discussion

The discriminative feature of the proposed model is the shape it takes. It starts from low number of channels, in the first block, then the number of channel increases while we go deeper. The feature map spatial resolution starts from the input image’s size then it became smaller while we go deeper in the network for simpler computations and better features learning. The smaller spatial resolution (down-sampling) is achieved through stride = 2 in some convolution operations but not everyone to avoid aggressive feature map spatial size reduction. This model shape is inspired from the networks such as VGG16, VGG19, Mobilenet, Shifflent and Xception networks. These networks are DAG networks, which have more complex architectures. The proposed model is light and simple, its architecture is linear so that each layer in the proposed model has a single input and each output goes to a single layer. Unlike DAG architecture, the proposed model is free of Addition/Concatenation operation and residual connections. Residual connections are not needed in the proposed model due to its simplicity and few number of layers.

The proposed architecture consists of five repeated building blocks based on 3 × 3 DWSC as in Fig. 4. It is a lightweight model and has roughly 664.6 Kilos learnable parameters. We compare the complexity of same model but based on Standard Convolution (SC) which has 3.8 million and found it is 5.7 × fewer learnable parameters than SC-Model. Assuming number of channels *N*, DWSC use number of learnable parameters = ${filterSize}^{2}$  × 1 × 1 x *N,* against ${filterSize}^{2}$ x ${N}^{2}$ learnable parameters in case the SC-model. This occurs at the same or small increase in accuracy compared with the same SC -model, as seen in Table 6. Therefore, in the proposed model (DWSC-model) provides competitive accuracy with much lower number of parameters compared with the SC-model and compared with other networks with millions learnable parameters. This is due to the main benefits of DWSC operation which is providing more efficient use of network parameters and an excellent trade-off between the model complexity and its representation capability^36,37^. The pointwise 1 × 1 convolution is simply a linear projection of exact same spatial size and the same number of channels; however, it allows the model to include extra non-linearity through the activation function without affecting the receptive field.

## Conclusion

The proposed lightweight CNN based on depth-wise separable convolution (DWSC) was tested on normal and different arrhythmic heartbeats and it was found effective to enhance the performance of person identification for several common arrhythmia types. The experimental results show excellent recognition performance on normal heartbeats (99.28%) as well as on arrhythmic heartbeats (93.81%). It outperformed other commonly used models in term of average accuracy as shown in Table 7. We generated ROC curves (Fig. 6) using the FAR and FRR for each class compared to the rest classes (one-versus-rest mode of classification). Although it is possible to balance the error rates for an authentication system by fine tuning the network parameters which we like to leave as a future work. As other possible future works, we also aim to investigate challenges such as automatic scalability due to increase of users, recognition with noisy signals, and effect of ECG signal variability on recognition performances due to different physical conditions such stress or exercise activities.

The model's scalability (i.e., adding more users/classes) is an important requirement of an identification system in real-world situations. Instead of requiring additional training of the entire model from scratch, the system should be able to automatically adopt itself. However, the automatic adaptation of an existing model presents a challenge and we would like to look into a few solutions for this as a future work. First, there have been some recent studies on Automated Machine Learning (AutoML)^48^, which could be incorporated into biometrics. Another alternative strategy is to use transfer-learning to retrain the previously trained model on the new pattern, after fine-tuning the old model by adjusting the final fully connected layer and the classification layer to account for the additional class. This technique could involve optimizing the model's hyperparameters for training on the new class using optimization algorithms such as Bayesian optimization for automated hyperparameter tuning algorithms. With the use of these methods, the model's performance on the new task can be enhanced by automatically finding the ideal hyperparameters without the need for human intervention. Finally, automated incremental learning (Online learning) for an adaptive deep learning framework^49^ could also be exploited to handle the scalability.

## Data Availability

The data supporting reported results presented in this study, MIT-BIH Dataset, are openly available in PhysioNet at https://physionet.org/content/mitdb/1.0.0/, reference number YYYY^28^.The analyzed and segmented data generated during this study can be found in: https://github.com/AwabedM/ECG-Biometrics/tree/main/Data. Where no new data were created.
